# Dual inhibition of MMP-2 and actin dynamics by a novel bis-chalcone: an anticancer strategy for oral squamous cell carcinoma

**DOI:** 10.1007/s00210-026-05050-0

**Published:** 2026-02-21

**Authors:** Rodrigo Elísio de Sá, Bruna Oliveira de Almeida, Marcelo da Costa Mota, Matheus Pedrosa de Oliveira, Anali Del Milagro Bernabe Garnique, Keli Lima, Aline Bernardes Valeze, Jefferson Almeida Rocha, Caridad Noda Pérez, João Agostinho Machado Neto, Letícia Veras Costa Lotufo, José Delano Barreto Marinho Filho, Ana Jérsia Araújo

**Affiliations:** 1 Laboratório de Cultura de Células do Delta (LCCDelta), Universidade Federal Delta do Parnaíba, UFDPar, Parnaíba, PI Brazil; 2 Departamento de Farmacologia, Instituto de Ciências Biomédicas, Universidade de São Paulo, USP, São Paulo, SP Brazil; 3 Departamento de Ensino, Instituto Federal de Educação, Ciência e Tecnologia de Mato Grosso - IFMT, Cuiabá, MT Brazil; 4 Laboratório de Sínteses Orgânica e Catálise, Instituto de Química, Universidade Federal de Goiás, UFG, Goiânia, GO Brazil; 5 Grupo de Pesquisa Em Química Medicinal E Biotecnologia (QUIMEBIO), Universidade Federal do Maranhão, UFMA, São Bernardo, MA Brazil

**Keywords:** Symmetrical bis-chalcone, Oral squamous cell carcinoma, Cell migration, Cell invasion, Metalloproteinases

## Abstract

Oral squamous cell carcinoma (OSCC), accounts for approximately 90% of oral cancer cases, and due to its aggressive nature and limited treatment options remains a significant clinical challenge. In the pursuit of novel therapeutic agents, bis-chalcones (a subclass of chalcones) have emerged as promising candidates with multipronged anticancer potential. In this study, we synthesized and evaluated a novel bis-chalcone (B2OCH_3_), for its cytotoxic, anti-migratory, anti-invasive, and anti-adhesive activities, as well as potential mechanisms of action. The compound was obtained via Claisen–Schmidt condensation and spectroscopically characterized. Cytotoxicity was assessed using MTT, pulse treatment, and trypan blue exclusion assays. Morphological changes were examined with panoptic staining and immunofluorescence. Clonogenic, migration, adhesion, and invasion assays were performed to assess anti-metastatic potential. Molecular docking and gene/protein expression analyses were conducted by quantitative PCR and Western blot. B2OCH_3_ exhibited potent cytotoxicity against HSC-3, SCC-4, and CAL-27 OSCC cell lines, with HSC-3 being the most sensitive. The compound demonstrated selectivity for cancer cells as compared with non-tumor HS5 and HaCaT cells. Reduced viability was accompanied by mitotic figures and apoptotic bodies. B2OCH_3_ significantly inhibited colony formation, migration, invasion, and adhesion in HSC-3 cells. Morphological analysis revealed modulation of actin filament organization and reduced filopodia formation. Molecular docking indicated MMP-2 and F-actin affinity, and was corroborated by molecular assays confirming protein modulation for both. Our findings identify B2OCH_3_ as a promising therapeutic candidate for OSCC, highlighting its potential to simultaneously target both MMP-2 activity and actin cytoskeleton regulation.

**Supplementary Information:**

The online version contains supplementary material available at 10.1007/s00210-026-05050-0.

## Introduction

Oral squamous cell carcinoma (OSCC) ranks as the sixth most prevalent cancer worldwide and constitutes a major subset of head and neck malignancies (Fatima et al. 2024). Epidemiological data from the Oral Cancer Foundation indicate a steadily rising incidence, with over 300,000 new cases diagnosed globally per year. Although significant progress has been achieved in current therapies, recurrence remains a frequent clinical event and continues to be strongly correlated with poor survival outcomes (Haque et al. 2019).

A central determinant of this prognosis is the metastatic potential of OSCC. Metastasis involves a series of biological processes through which malignant cells dissociate from the primary lesion, acquire migratory and invasive properties, penetrate vascular structures, disseminate systemically, and eventually establish secondary foci (Welch and Hurst 2019; He et al. 2025). The clinical impact is profound. Patients with metastatic OSCC exhibit a markedly reduced five-year survival rate of less than 6.3% (Sung et al. 2021). Effective treatment will require an understanding of the events that drive metastasis and the development of strategies to counteract the phenomenon.

Medicinal chemistry offers an avenue for the discovery of innovative compounds that can modulate metastatic behavior (Araújo et al. 2024) Chalcones constitute a class of compounds which exhibit a broad spectrum of biological activities against various diseases. Their chemical structure, characterized as a privileged scaffold, provides versatility for the design and development of new drugs (Zhuang et al. 2017). In addition, due to their ability to interact with multiple active sites, chalcones display affinity for various enzymes and molecular targets. Consequently, they have emerged as promising therapeutic agents, exhibiting antioxidant, anti-inflammatory, anti-neurodegenerative, anticancer, antiviral, and antiparasitic properties (Zhang et al. 2017; Constantinescu and Lungu 2021).

Our interest has been primarily directed toward bis-chalcones, compounds that contain two chalcone units within a single molecule. Bis-chalcones represent an extraordinary chemical scaffold with multifaceted pharmacological properties. Owing to this, they are considered highly attractive compounds and have been studied in a wide spectrum of potential biological applications (Olender et al. 2024). Numerous bis-chalcone derivatives have been investigated as antimicrobial, anticonvulsant, antidiabetic, antioxidant, antitubercular, anti-HIV, anti-amoebic, and anti-inflammatory agents, as well as their carbonic anhydrase and AChE inhibitor activity (Pereira et al. 2023). Chalcone-based structures have demonstrated anticancer activity (Modzelewska et al. 2006; Sharma et al. 2010; Fathi et al. 2021).

Though various bis-chalcone derivatives have been shown to possess anticancer activity, including inhibition of proliferation, colony formation, and even migratory/invasive properties (Burmaoglu et al., 2021; Nian et al., 2024), none have been specifically evaluated in OSCC. For example, boronic acid bis-chalcones display activity in breast cancer cells (Modzelewska et al. 2006). Asymmetric bis-chalcones inhibit colony formation and induce apoptosis via the FGFR1/ERK pathway in gastric cancer (Nian et al., 2024). Our study compound (B2OCH_3_) uniquely targets OSCC, affecting phenotypes linked to adhesion and migration.

In light of these activities, bis-chalcone scaffold-based molecules hold significant promise for applications in medicinal chemistry. Bis-chalcones appear to be potential candidates for the development of new drugs for the treatment of various diseases, including cancer. Although numerous studies have reported the synthesis of bis-chalcones as well as their diverse biological roles, a full understanding of their cytotoxic effects and their impact on processes associated with tumor metastasis remains limited. In this context, our study stands out as the first to systematically investigate the impact of a novel bis-chalcone (B2OCH_3_) on key events of metastatic behavior in HSC-3 cells. The present work aimed to synthesize and evaluate the effects of B2OCH_3_ and modulation of tumor aggressiveness, with emphasis on migration and adhesion processes, using both in vitro and in silico approaches.

## Materials and methods

### Synthesis and physicochemical characterization of bis-chalcone B2OCH_3_

**Fig. 1:**
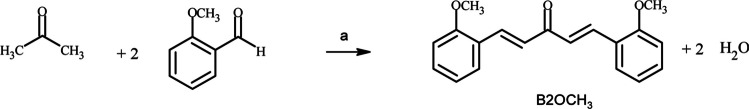
Synthesis of chalcone B2OCH_3_. (**a**) NaOH (6 M), MeOH, 15 min at room temperature

Bis-chalcone (B2OCH_3_) was synthesized by Claisen-Schmidt condensation as described previously by (Batovska et al. 2007), with slight modifications. The corresponding benzaldehyde (1.5 mmol) was dissolved in methanol (1 mL) and acetone (0.73 mmol), and 1.5 mL of 6 M NaOH solution was added. After 15 min of stirring at room temperature, the precipitate was filtered and crystallized from methanol. The Fig. 1 outlines chalcone synthesis.

The chemical structure of (1E,4E)−1,5-Bis(2-methoxyphenyl)−1,4-pentadien-3-one (B2OCH_3_) was confirmed by IR and ^1^H NMR spectral techniques. The melting point (M.p.) was measured, and elemental analyses (C, H) were performed.

(1E,4E)−1,5-Bis(2-methoxyphenyl)−1,4-pentadien-3-one (B2OCH_3_): Yellow crystals, yield 81%. M.p.: 119–120 ºC. IR (KBr) cm⁻^1^: 3031, 2840, 1670, 1615, 750. ^1^H NMR (500 MHz – CDCl₃) δ: 8.08 (d, 2H, J = 15.7 Hz, Hβ), 7.63 (dd, 2H, J = 7.7, 1.8 Hz, H6), 7.38 (ddd, 2H, J = 7.3, 1.7, 1.2 Hz, H5), 7.19 (d, 2H, J = 15.7 Hz, Hα), 7.00 (t, 2H, J = 7.7 Hz, H3), 6.94 (d, 2H, J = 8.4 Hz, H4), 3.92 (s, 6H, -OCH₃). Anal. Calcd. for C₁₉H₁₈O₃: C, 77.53; H, 6.16. Found: C, 78.1; H, 6.2%. A coupling constant of 15.7 Hz for the vinyl H-atoms confirmed the (E)-configuration (spectra provided in Supplementary Material, Figs. S1 and S2). The melting point, IR, and ^1^H NMR data are consistent with previously reported values (Adams et al. 2004; Liang et al. 2009).

### Cytotoxicity assay

The cytotoxic effects were evaluated using the MTT assay (Mosmann 1983; De Sá et al. 2023). Human oral squamous cell carcinoma (OSCC) cell lines HSC-3, SCC-4, and CAL-27 were used as cancerous models, whereas HS-5 (human bone marrow stromal cells) and HaCaT (immortalized human keratinocytes) served as non-cancerous control models. Cells in the exponential growth phase were plated in 96-well plates at a density of 7 × 10^4^ cells/mL.

After 24 h of incubation, the cells were exposed to B2OCH_3_ at concentrations ranging from 0.01 to 50 μM. The cells were then incubated at 37 °C for periods of 24, 48, and 72 h. Dimethyl sulfoxide (DMSO) served as the control. Following treatment, MTT solution (0.5 mg/mL) was added to each well and incubated for 3 h. The resulting MTT-formazan product, dissolved in DMSO, was quantified by measuring absorbance at 595 nm using a microplate reader. An intermittent exposure protocol was applied in which cells were treated with B2OCH_3_ for 3, 6, 12, or 24 h to evaluate the impact of different exposure durations on viability. Following compound removal, cells were incubated for a total of 72 h to capture both immediate and delayed effects on proliferation and survival (Almeida et al. 2019).

### Trypan blue

Cell viability was assessed using the trypan blue exclusion assay after incubating HSC-3 cells (7 × 10^4^ cells/mL) with cytotoxic (3.25 and 6.5 μM) and subcytotoxic (0.25 and 0.5 μM) concentrations of B2OCH_3_, as determined by the prior cytotoxicity assay. After 48 h, a 90 μL aliquot of the cell suspension was mixed with 10 μL of 0.4% trypan blue. Viable and nonviable cells were counted in a Neubauer chamber (De Sá et al. 2023).

### Morphological analysis

HSC-3 cells treated with B2OCH_3_ at concentrations of 3.25 and 6.5 μM for 24 h were examined under light microscopy. To observe morphological changes, after incubation, the cells were harvested, transferred onto cytocentrifuge slides, fixed with methanol for 30 s, and stained using the Rapid Panoptic staining kit to visualize the nucleus and cytoplasm. The cells were then analyzed under a light microscope at 400 × magnification.

### Clonogenic assay

HSC-3 cells were plated at a density of 3 × 10^2^ cells per well in sterile 6-well plates and incubated at 37 °C with 5% CO_2_ for 24 h. Following this incubation, subcytotoxic concentrations of B2OCH_3_ (0.25 and 0.5 μM) were applied to the wells and incubated for an additional 24 h. The cells were then washed with phosphate-buffered saline (PBS) and subsequently cultured in medium supplemented with 10% FBS for five more days under the same conditions. After this period, the medium was removed, and the cells were fixed with a methanol–acetic acid solution (3:1) for 5 min, followed by staining with a 2% crystal violet solution in methanol for 5 min.

### Anchorage-independent growth assay

In a 12-well plate, a 500 µL layer of 0.5% agarose was first added to establish the base layer. Another 500 µL was then used for the top layer, containing human oral squamous cell carcinoma cells, treated or untreated with 0.25 and 0.5 µM B2OCH_3_, suspended in 0.3% agarose. To prevent evaporation, 200 µL of medium was placed on top of the agarose layers. The plates were incubated for approximately 21 days to allow colony formation. Following this incubation, the colonies were stained with MTT solution (5 mg/mL) for 45 min. Images were captured and subsequently analyzed using image analysis software.

### Matrigel invasion assay

The migration potential of HSC-3 cells was evaluated using a Boyden transwell system with 8 μm pore-sized filters. After 12 h treatment with B2OCH_3_ (0.25 and 0.5 µM), cells were placed in the upper chamber, while the lower chamber was filled with 0.5 mL medium containing 20% FBS. Following an incubation period of either 24 or 10 h, the cells were stained with crystal violet. Any non-migratory cells remaining on the upper filter surface were carefully removed, and the cells that migrated to the underside of the filter were counted under a light microscope. For the invasion assay, transwell filters (8 μm pore size, 0.33 cm^2^ area) were coated with 50 μL of Matrigel diluted to 25 mg/50 mL using cold, filtered medium without FBS and allowed to polymerize at 37 °C before seeding the cells. After 48 h of incubation, the invading cells were stained and counted as described previously.

### Cell adhesion assay

The adhesion assay was performed with modifications based on the previously described method (Chen 2012). First, 10 μg/mL type I collagen was used to coat the wells for 1 h, followed by two washes with PBS and a blocking step using 1% bovine serum albumin (BSA) in PBS for 1 h. Cells were plated in 24-well plates at a concentration of 2 × 10^5^ cells/mL in serum-free DMEM and treated with B2OCH_3_ at 0.25 and 0.5 μM. After 2 h of incubation at 37 °C, the supernatant was carefully discarded, and the wells were rinsed twice with PBS. The cells were fixed using a 0.1% triarylmethane solution, stained with 0.1% xanthene and 0.1% thiazine, and the excess dye was removed by washing. The stained cells were then analyzed and imaged using an inverted optical microscope.

### Scratch assay

Approximately 9 × 10^4^ HSC-3 cells were plated in each well of a 24-well plate and allowed to grow until they reached 85–95% confluence. Afterward, the medium was removed, and a scratch was introduced at the base of each well using a 200 µL pipette. The wells were washed with PBS to eliminate debris, and fresh DMEM medium containing 0.25% FBS, along with either the vehicle (Ø) or varying concentrations of B2OCH_3_ (0.25 and 0.5 μM), were added. Images were captured at regular intervals (0, 24, and 48 h) using a digital inverted light microscope. Finally, the percentage of migration was calculated using the following formula: [(Scratch area _0 h_)—(Scratch area _24/48 h_)] × 100/(Scratch area _0 h_).

### Migration assay on spheroid model

Each well of a 96-well plate was prepared with 65 μL of 1% agarose at the bottom, and 1 × 10^4^ HSC-3 cells were seeded per well. The cells formed spheroids, which were cultured for 7 days. Once the spheroids were established, they were transferred to a 6-well plate. After the spheroids adhered to the surface, they were treated with either the vehicle (Ø) or various concentrations of B2OCH_3_ (0.25 and 0.5 μM). Periodic imaging was performed using a digital inverted light microscope.

### Immunofluorescence assay

The HSC-3 cells, treated with the vehicle (Ø) or 0.5 µM concentration for 3 and 6 h, were fixed using 100% cold methanol and made permeable with 0.5% Triton X-100 in PBS for 30 min at room temperature. Subsequently, the cells were blocked with 1% bovine serum albumin (BSA) in PBS for 1 h at room temperature. The cells were then incubated overnight at 4 °C with anti-α-tubulin conjugated to a fluorophore, in the dark. After washing once with PBS, the cells were stained with phalloidin conjugated to a fluorescent dye for 1 h. Following staining, the slides were mounted with an antifade mounting medium containing DAPI for 1 h at room temperature. Images were taken using a fluorescent microscope at × 400 magnification.

### Molecular docking

The 3D structures of the target proteins were retrieved from the Protein Data Bank (PDB) (https://www.rcsb.org/) (Berman et al. 2000), with the following PDB IDs: 2ZWH (F-actin), 8H78 (MMP-2), and 6ESM (MMP-9). Protein preparation was performed in Chimera v.13.1 (Pettersen et al. 2004) by removing water molecules and other groups, such as ions. The ligand 1,5-Bis(2-methoxyphenyl) penta-1,4-dien-3-one was obtained from PubChem (https://pubchem.ncbi.nlm.nih.gov/). Docking simulations were conducted using AutoDock Vina (Trott and Olson 2010), with ligands and proteins prepared in AutoDock Tools v.1.5.6 (Morris et al. 2008). Receptors were treated as rigid and ligands as flexible. Hydrogens were added, Gasteiger charges calculated, and non-polar hydrogens merged (Gasteiger and Marsili 1980). Method validation was performed by re-docking crystallographic ligands, yielding RMSD ≤ 2.0 Å using PyRx (Ferrari and Patrizio 2021; Repasky et al. 2012). Docking poses and overlap between co-crystallized and re-docked ligands were analyzed in Discovery Studio 2.0. The x, y, and z coordinates of the computational poses defined the active site region (Table 1 of the supplementary material). Docking parameters included 50 modes, exhaustiveness of 24, and a grid box size of 30 points per axis. The best binding energy values (ΔGbind) were used to assess interaction efficiency (Rocha et al. 2018).

### Quantitative PCR

Total RNA from HSC-3 cells treated with B2OCH3 (0.5 μM) or a negative control was extracted, and cDNA was synthesized from 1 µg of RNA. Quantitative PCR (qPCR) was performed with specific primers for GAPDH, MMP-2, and MMP-9. Primer sequences are listed in Table 2 of the supplementary material. Relative quantification was calculated using the 2^−ΔΔCT^ method (Livak and Schmittgen 2001).

### Western blot analysis

Protein extraction was performed from HSC-3 cells treated with vehicle or B2OCH3 (0.25 and 0.5 µM) for 48 h. Protein concentration was determined using the Bradford assay. Equal amounts of protein (30 μg) were separated on SDS-PAGE gels and transferred onto membranes. Primary antibodies against GAPDH, Pan-actin, and MMP-2 were incubated overnight at 4 °C, followed by HRP-conjugated secondary antibodies for 2 h at room temperature. Detection was performed using a chemiluminescent substrate, and bands were visualized with a gel imaging system.

### Statistical analysis

Statistical analyses were performed, and IC_50_ values were obtained by non-linear regression. The results of three independent experiments are represented as the mean ± standard error of the mean (S.E.M). Statistical comparisons were made using one-way analysis of variance (ANOVA) followed by Tukey's post-test, with significance defined as *p < 0.05.

## Results

### Cytotoxicity of B2OCH_3_ and its impact on colony formation of HSC-3 cells

**Fig. 2:**
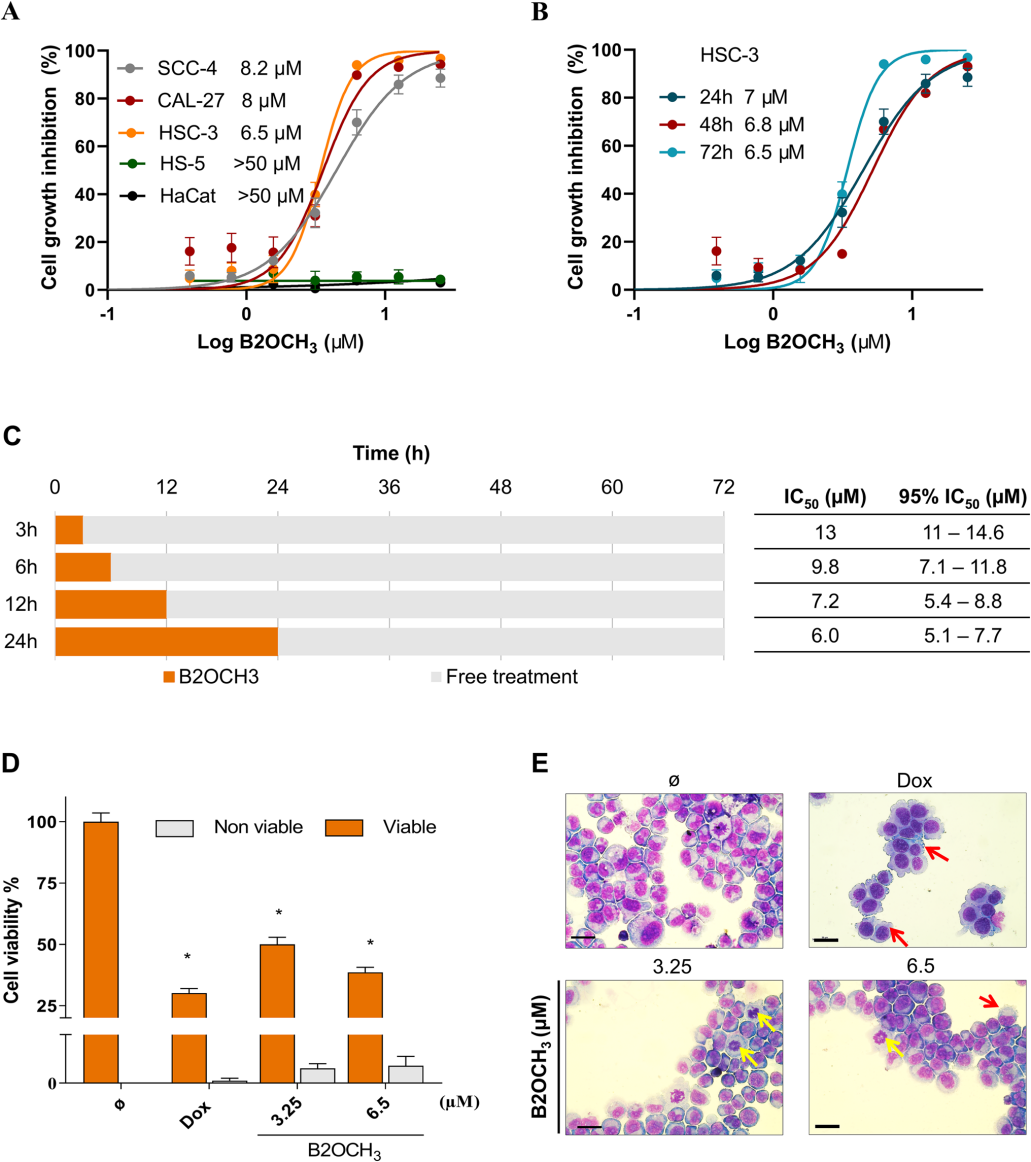
Cytotoxic activity of B2OCH_3_ in non-tumor and tumor cells. **A**) According to the MTT assay, B2OCH_3_ reduces cell viability in tongue squamous cell carcinoma cell lines (HSC-3, SCC-4, and CAL-27) and shows selectivity toward non-tumor stromal (HS-5) and keratinocyte (HaCaT) cells. Dose–response curves of cytotoxicity kinetics after 72 h of treatment (*n* = 3 independent experiments, each performed in triplicate). **B**) Dose–response curves for B2OCH3 treatment for 24, 48, and 72 h in HSC-3 cells, assessed by the MTT assay (*n* = 3 independent experiments, each performed in triplicate). **C**) Intermittent exposure (pulse) assay. The bar graph illustrates the exposure schedule of HSC-3 cells to B2OCH_3_ (orange bars), followed by incubation periods in drug-free medium (gray bars), designed to evaluate the effects of transient drug exposure on cell recovery and viability Dose–response curves for B2OCH3 treatment for 24, 48, and 72 h in HSC-3 cells, assessed by the MTT assay (*n* = 3 independent experiments, each performed in triplicate). **D**) Percentage of viable HSC-3 cells in control and B2OCH3-treated groups (3.25 and 6.5 µM), determined by the trypan blue exclusion assay (*n* = 3 independent experiments, each performed in triplicate). **E**) Optical microscopy of HSC-3 cells after 24 h of incubation with B2OCH_3_ (3.25 and 6.5 µM). Red arrow: bleb formation; yellow arrow: mitotic figures. Scale bar 20 μm. Data are expressed as mean ± SEM. IC₅₀ values were calculated by nonlinear regression using GraphPad Prism software (version 8.0), and 95% confidence intervals (95% CIs) were obtained from at least three independent experiments performed in triplicate. Statistical significance was determined by one-way ANOVA followed by Tukey’s multiple comparisons test (*p < 0.05)

Initially, the cytotoxic effects of B2OCH_3_ were evaluated in human oral squamous cell carcinoma and stromal cells at 24, 48, and 72 h of incubation. As shown in Fig. 2A and B, B2OCH_3_ reduced tumor cell viability under all tested conditions, with no additional increase in cytotoxicity after 24 h. The IC_50_ values calculated ranged from 6.5 to 7.0 µM (Fig. 2B). In HS5 and HaCaT cells, no toxicity was observed (IC_50_ exceeding the highest tested concentration of 50 µM), indicating that B2OCH_3_ exhibits selectivity toward HSC-3 cells (Fig. 2B). Doxorubicin, used as positive control, presented significant toxicity against HSC-3 cells (IC_50_ ranging from 0.21 to 0.29 µM) (Table 3 of the supplementary material), being 24 times more potent than B2OCH_3_ at 24 h incubation. However, doxorubicin seemed less selective than the bis-chalcone, as it was also toxic to stromal cells. The selectivity index (IC_50_ for stromal cells/IC_50_ for oral carcinoma cells) for doxorubicin after 24 h of incubation was 2.14. For B2OCH_3_, the selectivity index was estimated at 7.14 using the highest tested concentration in stromal cells (50 µM) (Fig. 3 of the supplementary material), as it was not possible to determine the IC_50_ values.

**Fig. 3:**
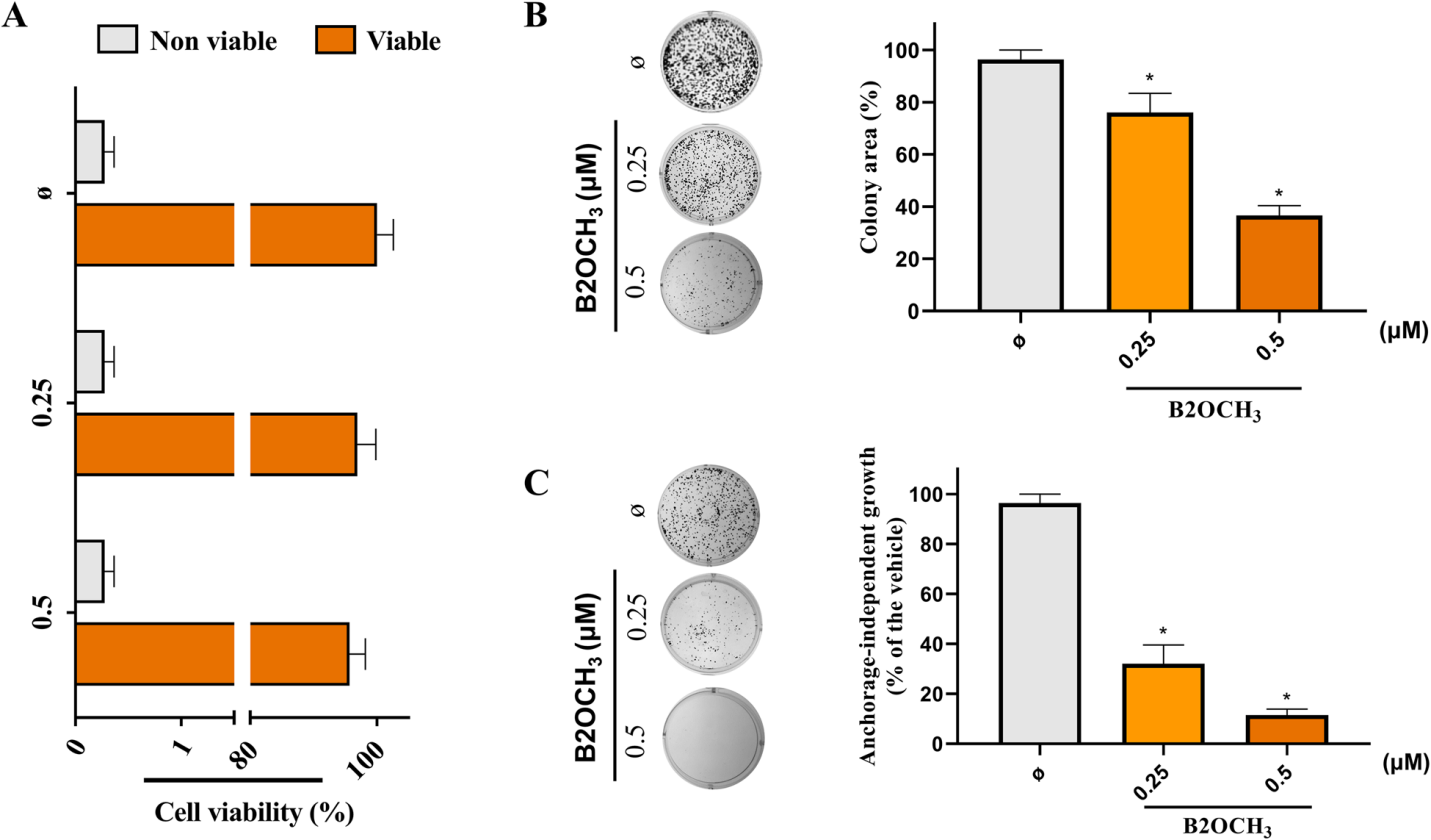
Effects of subcytotoxic concentrations of B2OCH_3_ on HSC-3 cells. **A**) Percentage of viable HSC-3 cells in control and B2OCH_3_-treated groups (0.25 and 0.5 µM), assessed by Trypan blue exclusion assay (*n* = 3 independent experiments, each performed in triplicate). **B**) Effect of B2OCH_3_ on colony formation in HSC-3 cells; representative image from triplicates and corresponding quantification of colony area (*n* = 3 independent experiments). **C**) Anchorage-independent growth of HSC-3 cells treated with vehicle or B2OCH_3_ for 21 days in soft agar. Bar graphs show mean ± standard error of the mean (SEM) from three independent experiments (*n* = 3 independent experiments). Bar graphs show mean ± SEM. Statistical significance was determined by one-way ANOVA followed by Tukey’s multiple comparisons test (*p < 0.05). Effect size is expressed as percentage inhibition relative to the control

A 3 h pulse treatment with B2OCH_3_ was sufficient to significantly decrease cell viability measured after a total incubation time of 72 h (Fig. 2C). When the total incubation time was reduced to 24 h (even with continuous exposure to the drug), the cytotoxic effects were reduced (Fig. 2C). Trypan blue staining results also demonstrated a significant reduction in the total number of cells after treatment with B2OCH_3_ (Fig. 2D). Morphological analysis with rapid panoptic staining revealed the presence of mitotic figures, and a reduction in cell density and cell bodies at a concentration of 6.5 μM after treatment with B2OCH_3_ (Fig. 2E).

As B2OCH_3_ induced marked reductions in cell viability after only 3 h of exposure, with sustained effects even after 72 h in drug-free medium, and morphological analyses revealed changes consistent with mitotic arrest, we hypothesized that these effects might not be restricted to direct cytotoxicity. We therefore investigated whether these effects might also impact functional events related to tumor progression, such as cell migration and adhesion.

Sub-cytotoxic concentrations of 0.25 and 0.5 µM were selected based on the IC_50_ values for subsequent experiments with the bis-chalcone, ensuring minimal cell death and allowing a clearer assessment of the biological response. According to literature reports, concentrations below the IC_50_ values are commonly employed in in vitro studies with structurally related chalcones, reinforcing the experimental suitability and biological relevance of these conditions (Jansson-Löfmark et al. 2020; Araújo et al. 2024). The trypan blue exclusion assay confirmed that neither concentration selected affected HSC-3 cell viability (Fig. 3A), and clonal growth was inhibited by exposure to B2OCH_3_ at both concentrations tested. B2OCH_3_ reduced the colony area by 24% at 0.25 μM and by 63.4% at 0.5 μM (Fig. 3B). In the anchorage-dependent assay, the compound suppressed colony formation by 68% at 0.25 μM and by 88.5% at 0.5 μM (Fig. 3C).

### Effects of B2OCH_3_ on stages of tumor progression and cell morphology

**Fig. 4:**
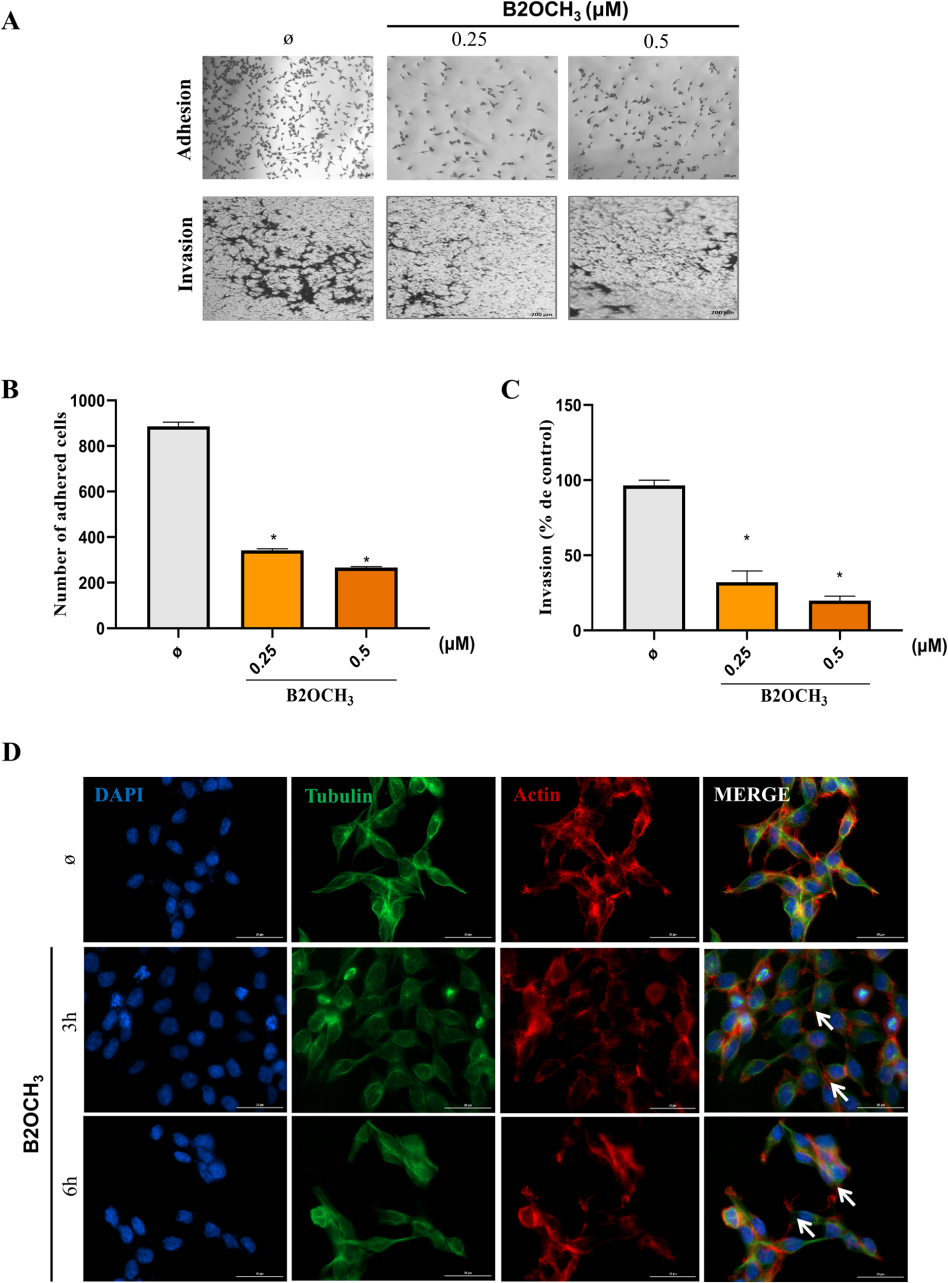
Effects of B2OCH₃ on adhesion, invasion, and cytoskeletal alterations in HSC-3 cells. **A**) Representative images of HSC-3 cell adhesion to type I collagen and invasion assay (crystal violet staining). **B**) Quantification of cell adhesion to type I collagen, based on two representative fields per replicate (3 independent experiments, each performed in triplicate). **C**) Quantification of cell invasion, expressed as a percentage relative to the control group (3 independent experiments, each performed in triplicate). **D**) Immunofluorescence images showing nuclei (DAPI, blue), microtubules (α-tubulin, green), and actin filaments (F-actin, red) after 3 and 6 h of exposure to B2OCH₃ (0.5 µM). Arrows indicate cytoskeletal changes. For each condition, four quadrants per well were photographed and analyzed. Data are expressed as mean ± SEM. Statistical significance was determined by one-way ANOVA followed by Tukey’s multiple comparisons test (*p < 0.05)

To investigate whether B2OCH_3_ influences tumor progression-related events in HSC-3 cells, we performed adhesion, invasion, and migration assays. Treatment with B2OCH_3_ at sub-cytotoxic concentrations (0.25 and 0.5 μM) significantly inhibited adhesion of HSC-3 cells to type I collagen (Fig. 4A). Specifically, 0.25 μM and 0.5 μM B2OCH_3_ reduced adhesion to 350.9 ± 35.0 and 270.8 ± 30.0 cells per field, respectively, as compared with 900.2 ± 35.3 cells per field for the control group (Fig. 4B). Morphological alterations were observed in cells exposed to these concentrations as well.

Given that invasion is a crucial factor in cancer progression and metastasis, we investigated whether the compound B2OCH_3_ affected cancer cell invasion using Matrigel® coated Transwell® chambers. Consistent with the data from adhesion and migration assays, B2OCH_3_ significantly inhibited the invasion of oral squamous cell carcinoma (Fig. 4A). In HSC-3 cells treated with 0.25 and 0.5 μM B2OCH_3_, the invasion ratio relative to the control group was respectively reduced to 24% and 22% (Fig. 4C). These findings demonstrate that B2OCH_3_ significantly suppressed the invasive capacity of HSC-3 cells through Matrigel® in a dose-dependent manner.

To further investigate the effects of B2OCH_3_ on cellular processes associated with tumor progression, immunofluorescence analysis was performed to assess whether exposure to non-cytotoxic concentrations of the compound induces alterations in cell morphology. Untreated HSC-3 cells exhibited an elongated morphology with cytoplasmic extensions, indicating adhesion to the coverslip, along with a typical interphase microtubule network spanning the entire cell (Fig. 4D). In contrast, B2OCH_3_-treated cells displayed a rounded morphology with shrunken cell bodies, suggesting impaired adhesion and spreading ability. The treatment modified the cytoskeletal organization by altering the architecture of actin filaments and microtubules, resulting in pronounced cell retraction, as indicated by the white arrows. Notably, a reduction in filopodia formation was observed in the treated groups (Fig. 4D).

**Fig. 5:**
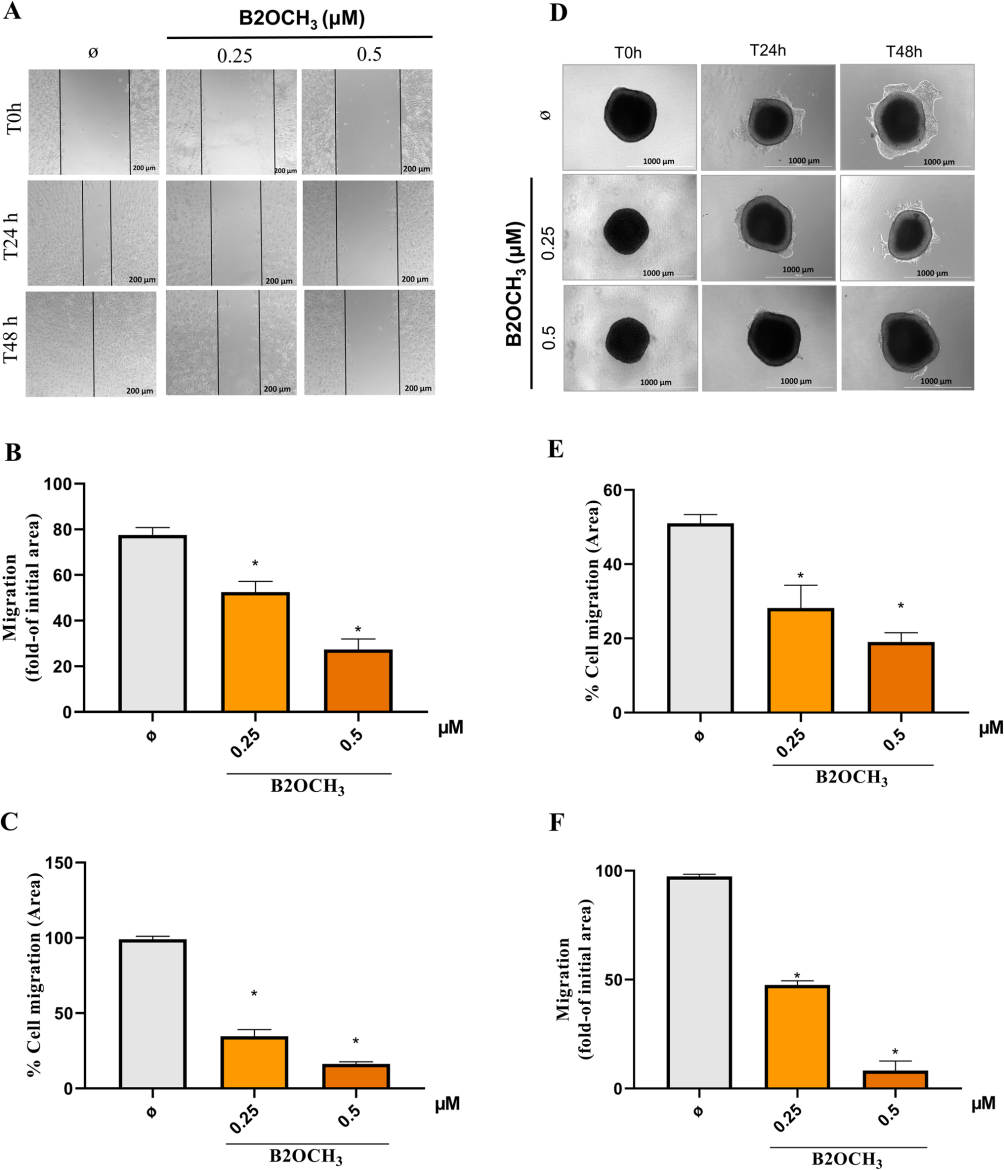
**A**) Representative images of the scratch wound-healing assay showing HSC-3 cell migration after treatment with vehicle or B2OCH₃ (0.25 and 0.5 μM) at 0, 24, and 48 h. Scale bar: 200 μm. Quantification of scratch closure after 24 h (**B**) and 48 h (**C**) of treatment. **D**) Representative images of the spheroid-based migration assay of HSC-3 cells treated with vehicle or B2OCH₃ (0.25 and 0.5 μM) for 24 and 48 h. Scale bar: 1000 μm. Quantification of the spheroid model after 24 h (**E**) and 48 h (**F**) of treatment. Data are expressed as mean ± SEM from three independent experiments (n = 3, each performed in triplicate). Statistical significance was determined by one-way ANOVA followed by Tukey’s multiple comparisons test (*p 0.05)

The scratch closure on the cell monolayer was calculated based on the scratched area at time zero and at 24 and 48 h of exposure to either vehicle or treatment (Fig. 5A). The compound at concentrations of 0.25 and 0.5 μM, significantly reduced the migration percentage compared to the negative control in the HSC-3 cell line. The results demonstrated that after 24 h of incubation, the percentage of cell migration when exposed to B2OCH_3_ was 24% at 0.25 μM and 22% at 0.5 μM, while untreated cells presented a percentage of 48% (Fig. 5B). The results also revealed that cells treated with 0.25 µM and 0.5 µM exhibited respective migration rates of 38% and 11.5% after continuous incubation for 48 h (Fig. 5C). The negative control presented a migration rate of 96.2% (Fig. 5C).

Cell migration was also assessed using a three-dimensional model of HSC-3 cells, and revealed that B2OCH_3_ treatment inhibits spheroid cell migration (as compared to the negative control spheroids) (Fig. 5D, E and F).

### B2OCH_3_ promotes interaction and changes in molecular markers involved in tumor progression

To explore the potential molecular interactions of B2OCH_3_, we conducted in silico analyses focusing on proteins implicated in metastatic progression, including actin and matrix metalloproteinases. These analyses served as a hypothesis-generating approach, with subsequent experimental validation prioritizing targets showing functional modulation.

Molecular docking analysis revealed favorable binding of B2OCH_3_ to actin (A), MMP-2 (B), and MMP-9 (C), with predicted binding free energies of − 7.7, − 8.3, and − 6.5 kcal·mol⁻^1^, respectively. In all complexes, B2OCH_3_ occupied well-defined binding pockets and established stabilizing interactions with residues located in functionally relevant regions of each protein.

**Fig. 6:**
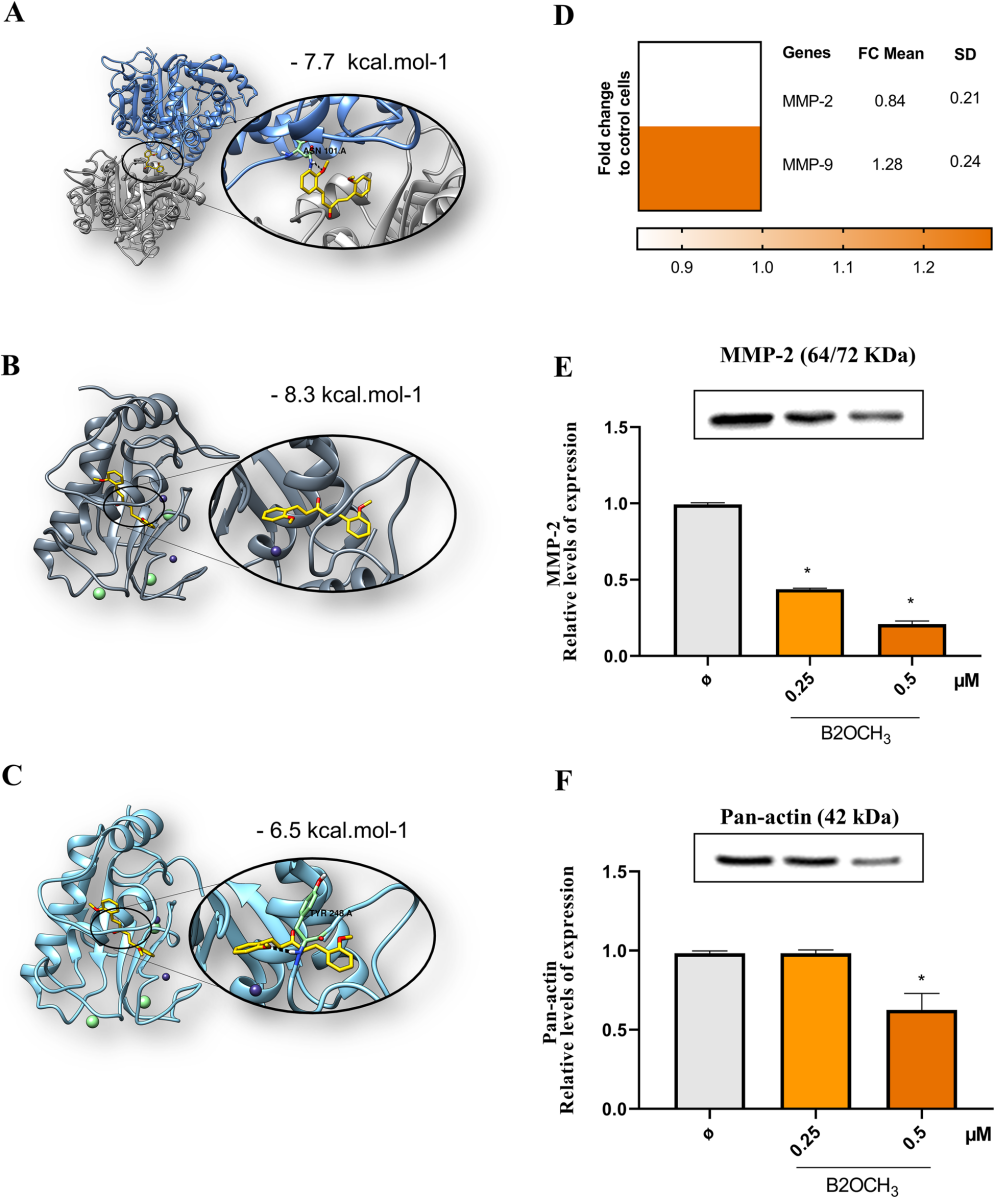
Molecular interactions of B2OCH_3_ with actin, MMP-2, and MMP-9, and their effects on gene and protein expression. (**A**-**C**) Tree and two-dimensional representations of the molecular interactions between the compound B2OCH₃ and the proteins actin (**A**), MMP-2 (**B**), and MMP-9 (**C**), indicating the binding site, molecular interactions and binding energy values (kcal mol⁻^1^) for each molecular complex. **D**) Heat map showing the relative variation in MMP-2 and MMP-9 gene expression in HSC-3 cells treated with B2OCH₃. Gene expression analysis was performed using samples from three independent experiments (*n* = 3). Orange shades indicate increased mRNA levels, while lighter shades indicate decreased expression. (**E–F**) Protein levels of pan-actin (**E**) and MMP-2 (F) in HSC-3 cells treated with B2OCH_3_. Membranes were reprobed with the antibody to detect total protein GAPDH. Quantitative data are expressed as mean ± SEM from three independent experiments (*n* = 3), each experiment performed in triplicate. Statistical significance was determined by one-way ANOVA followed by Tukey’s multiple comparisons test (*p < 0.05)

Among the evaluated targets, MMP-2 exhibited the strongest predicted interaction, which was subsequently corroborated by experimental evidence showing reduced mRNA (Fig. 6D) and protein levels following B2OCH_3_ treatment (Fig. 6E). Actin also displayed a favorable docking profile and was experimentally validated by decreased protein expression and marked disruption of the actin cytoskeletal network, as demonstrated by Western blot (Fig. 6F and immunofluorescence analyses (Fig. 4D).

Although MMP-9 showed a thermodynamically favorable binding energy in silico, its gene expression was not significantly modulated under the experimental conditions tested (Fig. 6D). Overall, these results support MMP-2 and actin as the primary experimentally validated molecular targets of B2OCH_3_.

When complexed with actin, hydrogen bond interactions were observed with Asn101(A), and hydrophobic bonds with Ala180(A), Thr179(A), Val181(A), Asn258(B), Met259(B), Lys352(B), Leu255(B), Ala250(B), Lys254(B), Leu248(B), and Asn249(B), as shown in (Fig. 5A of the supplementary material). In MMP-2, B2OCH_3_ interacts through hydrophobic contacts with (A), Val118(A), Tyr143(A), His121(A), Leu117(A), Thr146(A), Phe149(A), Ile142(A), Ala140(A), Thr144(A), Pro135(A), Ala137(A), Pro141(A), and Leu83(A) (Fig. 5B of the supplementary material). In MMP-9, a hydrogen bond was found at Tyr248(A), and other additional bonds at Ala189(A), Gln227(A), Arg249(A), Val223(A), Tyr245(A), His226(A), Met247(A), Ala242(A), Leu188(A), Leu243(A), Glu241(A), Thr251(A), Pro255(A), Leu222(A), Gly186(A), and Leu187(A) for MMP-9 (Fig. 5C of the supplementary material).

Taken together, these findings provide experimental support for MMP-2 as a primary molecular target and indicate modulation of actin-associated cytoskeletal organization.

## Discussion

This study describes the biological effects of a novel symmetrical bis-chalcone (B2OCH_3_) in in vitro models of oral squamous cell carcinoma (OSCC). Consistently, our findings indicate that (1) B2OCH_3_ exhibits potent cytotoxic activity against the HSC-3, SCC-4, and CAL-27 tumor cell lines, with remarkable selectivity over the non-tumoral HS5 cell line; (2) it significantly reduces the clonogenic potential and anchorage-independent growth of HSC-3 cells; (3) it impairs migration and invasion in both 2D and 3D models, in addition to inhibiting cell adhesion; and (4) at the molecular level, the compound decreases MMP-2 expression and reduces pan-actin protein levels. Taken together, these results support B2OCH_3_ as a promising preclinical lead compound with the potential to advance into studies employing more complex biological models. Although the available data are derived primarily from in vitro and in silico approaches, they provide a solid foundation for subsequent investigations in more advanced experimental systems.

In the context of current therapies for oral OSCC, the in vitro selectivity index enables comparison with chemotherapeutic agents commonly used in clinical practice, such as cisplatin, 5-fluorouracil, paclitaxel, and docetaxel, which typically display low micromolar IC₅₀ values in tumor cells but also affect non-tumor cells under in vitro conditions (Eloraby et al. 2024; Sati et al. 2024; Cabral et al. 2025). Within this framework, B2OCH_3_ exhibited a differential response between tumor and non-tumor cells across the tested concentration range. Importantly, the effective subcytotoxic concentrations fall within ranges reported for compounds at early preclinical stages; however, their pharmacological relevance remains to be validated in more complex biological models.

In HSC-3 cells, the effects occurred after a 3 h pulse treatment (at minimum) and lasted for several days, as demonstrated by the MTT assays (72 h). While speculative, this observation may provide initial insights into pharmacokinetic behavior, however this underscores the need for dedicated in vivo studies (De Almeida et al. 2019).

Previous studies have reported antiproliferative effects of bis-chalcone analogs in cancer cells (Yang et al. 2020; Abubakar et al. 2024). In line with these observations, B2OCH_3_ exhibited greater selectivity toward HSC-3 cells than toward the non-cancerous HS5 cell line, supporting its evaluation as a selective scaffold for therapeutic development. Based on these findings, we further investigated the effects of this compound on cancer cell biology. Among the established hallmarks of cancer, the ability to invade and metastasize is particularly critical, as metastatic disease remains a major cause of cancer-related morbidity and mortality (Hanahan 2022; Gerstberger et al. 2023).

Tumor metastasis is a complex, multifactorial process involving cell adhesion, invasion, and motility. For anti-metastatic therapy, targeting and disrupting any of these steps is a promising strategy (Pearson 2019). The capacity of cancer cells to form colonies reflects their ability to survive independently and adapt to diverse environmental conditions, offering insights into their tumorigenic potential following metastasis (Lambert et al. 2024). In this study, B2OCH_3_ was found to reduce the size of HSC-3 colonies, even at sub-cytotoxic concentrations. This finding is particularly noteworthy given the highly aggressive nature of HSC-3 cells, which are widely used to evaluate compounds targeting cell migration and invasiveness. Further, B2OCH_3_ effectively inhibited anchorage-independent colony formation in the soft agar assay. Resistance to anoikis, a form of apoptosis induced by detachment from the extracellular matrix, is a critical adaptation that facilitates cancer cell dissemination and represents a key process in metastatic cancers, including advanced tongue squamous cell carcinoma (Adeshakin et al. 2021).

During the functional analyses, B2OCH_3_ significantly inhibited both the migration and invasion of tongue cancer cells in 2D and 3D cellular models, primarily through modulation of key proteins involved in tumor progression (including actin and MMP-2). These processes require dynamic alterations in cell morphology, migratory capacity, adhesion to the extracellular matrix, and the ability to invade surrounding tissues. By disrupting these mechanisms, B2OCH_3_ may impair critical cellular processes associated with metastatic behavior in vitro (Hamidi and Ivaska 2019; Lima et al. 2023; Crossley et al. 2024). Successful cell migration depends on the coordinated assembly of adhesion structures and the organization of actin filaments. Evidence indicates that actin stress fiber organization is critical for cell stiffening during invasion and also contributes to the expansion of premalignant cancer cells (Mancini et al. 2024).

Immunofluorescence analysis showed that B2OCH_3_ treatment led to disorganization of the actin cytoskeleton and a reduction in cell body length in tongue cancer cells. These changes were associated with altered adhesion and reduced migratory and invasive behavior in vitro, particularly in the HSC-3 cell line, which represents a highly aggressive tongue cancer subtype. The observed decrease in adhesion, migration, and invasion may be related to metalloproteinase activity, specifically the binding and negative regulation of MMP-2 following exposure to B2OCH_3_. MMP activity is controlled by a complex network of molecular pathways within the tumor microenvironment and is influenced by multiple signaling molecules (Rajendran 2024; Shi et al. 2024). Consistent with these findings, inhibitory effects on MMP-2 have also been reported for other chalcone-based compounds in in vitro models (De Souza et al. 2022). Given the central role of matrix metalloproteinases in tumor progression, targeting MMP-2 interactions with cell surface molecules remains an area of active therapeutic interest (Lenci et al. 2021; Kim et al. 2023).

Although B2OCH_3_ exposure was associated with marked cytoskeletal disorganization and reduced formation of actin-based protrusions, the present data do not establish a direct cause–effect relationship between actin modulation and the impaired migratory and invasive behavior. Actin remodeling may represent a downstream consequence of altered cell–ECM interactions and reduced MMP-2 expression, both of which are known to influence focal adhesion dynamics and cytoskeletal organization (Eren et al. 2022) Alternatively, actin-associated pathways may be directly sensitive to B2OCH_3_, contributing to the observed phenotype. Given the absence of direct target engagement or rescue experiments, these mechanisms cannot be distinguished in the current study. Therefore, actin modulation is interpreted as a biologically relevant correlate of reduced migration rather than as a confirmed primary molecular target.

Our results demonstrated that B2OCH_3_ treatment markedly reduced filopodia formation, a feature we associated with decreased motility in HSC-3 cells. Immunofluorescence analysis also indicated that this loss of filopodia is likely linked to changes in adhesion dynamics and cytoskeletal rearrangements. Given the central role of filopodia in cytoskeletal organization, their loss is expected to influence cell architecture and contribute to the observed phenotypic changes (Janiszewska et al. 2020).

In addition to these structural changes, a clear shift in cell morphology was observed. While untreated HSC-3 cells displayed a spindle-like phenotype, B2OCH_3_-treated cells exhibited a more rounded shape. Cell morphology is intrinsically associated with the underlying cytoskeletal framework and biophysical properties of cells, including their mechanical characteristics (Zhao et al. 2020). Notably, metastatic cancer cells from various tumor origins often present cytoskeletal remodeling, which enhances their deformability and promotes invasive behavior. Thus, the morphological changes observed following B2OCH_3_ treatment may reflect biomechanical alterations associated with disrupted cytoskeletal organization and modulation of adhesion protein expression (Xiao et al. 2019; Li et al. 2021).

Reductions in focal adhesion are not only associated with integrin inhibition but may also reflect alterations in downstream signaling pathways mediated by the extracellular matrix (ECM) (Pang et al. 2023). In this study, B2OCH_3_ was associated with decreased pan-actin expression in HSC-3 cells. The actin cytoskeleton is known to contribute to cell motility through the formation of dynamic structures such as lamellipodia and filopodia. Taken together, our in silico and in vitro data suggest that modulation of actin-related cytoskeletal organization may be associated with the reduced migratory behavior observed after B2OCH₃ exposure.

This study employed an experimental in vitro model of metastatic tongue squamous cell carcinoma to investigate key aspects of cancer cell biology. The results demonstrate that B2OCH_3_ induces morphological, molecular, and functional alterations in HSC-3 cells under controlled experimental conditions. Specifically, the compound exhibited selective activity in vitro, impaired tumor cell growth, and reduced adhesion, migration, and invasion, concomitant with modulation of MMP-2 expression and actin-associated cytoskeletal organization.

Despite the strength and coherence of the in vitro findings, this study has inherent limitations, as all analyses were restricted to cellular models. Nevertheless, the results provide valuable insights and reinforce the potential relevance of B2OCH_3_, supporting further investigation into its safety, pharmacological profile, and pharmacokinetic behavior.

## Conclusion

Our study provides evidence that B2OCH_3_ exerts multiple biological effects in an in vitro model of tongue squamous cell carcinoma, leading to reduced cell viability and impairment of cellular processes associated with the metastatic phenotype, accompanied by morphological and molecular alterations. The data indicate that B2OCH₃ displays a selective profile and modulates pathways related to MMP-2 expression and actin-associated cytoskeletal organization, as supported by gene expression, protein analyses, and cytoskeletal imaging.

Overall, these findings demonstrate that B2OCH_3_ influences key cellular mechanisms associated with oral squamous cell carcinoma aggressiveness in experimental cell models.

## Supplementary Information

Below is the link to the electronic supplementary material.

Supplementary file1 (DOCX 6464 KB)

## Data Availability

All source data for this work (or generated in this study) are available upon reasonable request.

## Abbreviations

OSCC: Oral squamous cell carcinoma
MMP: Matrix metalloproteinase
DMEM: Dulbecco’s modified eagle medium
FBS: Fetal bovine serum
DMSO: Dimethyl sulfoxide
PBS: Phosphate-buffered saline
BSA: Bovine serum albumin
MTT: 3-(4,5-Dimethylthiazol-2-yl)-2,5-diphenyltetrazolium
PDB: Protein data bank
qPCR: Quantitative polymerase chain reaction
PMSF: Phenylmethylsulfonyl fluoride
EDTA: Ethylenediaminetetraacetic acid
SDS-PAGE: Sodium dodecyl sulfate–polyacrylamide gel electrophoresis
RMSD: Root mean square deviation

## References

[CR1] Abubakar S, Khairuddean M, Ismail NZ, Salhimi SM, Al-Amin M, Wahyuningsih TD (2024) Synthesis and computational insights of hybrid heterocyclic bis-chalcone compounds and their cytotoxic effects against breast cancer cells. Res Chem 101464. 10.1016/j.rechem.2024101464.

[CR2] Adams BK, Ferstl EM, Davis MC, Herold M, Kurtkaya S, Camalier RF, Hollingshead MG, Kaur G, Sausville EA, Rickles FR, Snyder JP, Liotta DC, Shoji M (2004) Synthesis and biological evaluation of novel curcumin analogs as anti-cancer and anti-angiogenesis agents. Bioorg Med Chem 12:3871–3883. 10.1016/j.bmc.2004.05006 15210154 10.1016/j.bmc.2004.05.006

[CR3] Adeshakin F, Adeshakin AO, Afolabi LO, Yan D, Zhang G, Wan X (2021) Mechanisms for modulating anoikis resistance in cancer and the relevance of metabolic reprogramming. Front Oncol 11:626577. 10.3389/fonc.2021.626577 33854965 10.3389/fonc.2021.626577 PMC8039382

[CR4] Almeida LC, Bauermeister A, Rezende-Teixeira P, Santos EAD, Moraes LAB, Machado-Neto JA, Costa-Lotufo LV (2019) Pradimicin-IRD exhibits antineoplastic effects by inducing DNA damage in colon cancer cells. Biochem Pharmacol 47:38–47. 10.1016/j.bcp.2019.06.016 10.1016/j.bcp.2019.06.016 31228463

[CR5] Araújo GS, Moura AF, Barros AB, Moraes MO, Pessoa C, Perez CN, Castro MRC, Ribeiro FOS, Silva DAD, Sousa PSA, Rocha JA, Marinho Filho JDB, Araujo AJ (2024) Sulfonamide-chalcone hybrid compound suppresses cellular adhesion and migration: experimental and computational insight. Chem Biol Interact 1:398–111115. 10.1016/j.cbi.2024111115 10.1016/j.cbi.2024.111115 38908811

[CR6] Bastrakov M, Starosotnikov A (2022) Recent progress in the synthesis of drugs and bioactive molecules incorporating nitro (het) arene core. Pharmaceuticals (Basel). 15:705. 103390/ph15060705 10.3390/ph15060705 PMC9228974 35745627

[CR7] Batovska D, Parushev S, Slavova A, Bankova V, Tsvetkova I, Ninova M, Najdenski H (2007) Study on the substituents effects of a series of synthetic chalcones against the yeast *Candida albicans*. Eur J Med Chem 42:87–92. 10.1016/j.ejmech.2006.08012 17007965 10.1016/j.ejmech.2006.08.012

[CR8] Berman HM, Westbrook J, Feng Z, Gilliland G, Bhat TN, Weissig H, Shindyalov IN, Bourne PE (2000) The protein data bank. Nucleic Acids Res 28:235–242 10592235 10.1093/nar/28.1.235 PMC102472

[CR9] Burmaoglu S, Gobek A, Ozturk Aydin B, Yurtoglu E, Nur Aydin B, Yalcin Ozkat G, Hepokur C, Simsek Ozek N, Aysin F, Altundas R, Algul O (2021) Design, synthesis and biological evaluation of novel bischalcone derivatives as potential anticancer agents. Bioorg Chem 109:104882. 10.1016/j.bioorg.2021.104882 10.1016/j.bioorg.2021.104882 33839582

[CR10] Cabral LGS, Oliveira CS, Oliveira VX Jr, Paulo EPA, Poyet J-L, Maria DA (2025) Evaluation of the antitumor and antiproliferative potential of synthetic peptides derived from IsCT1, associated with cisplatin, in squamous cell carcinoma of the oral cavity. Molecules 30(12):2594. 10.3390/molecules30122594 10.3390/molecules30122594 PMC12196148 40572557

[CR11] Chen Y (2012) Cell adhesion assay. Bio-Protoc 2:7–9. 10.21769/BioProtoc98

[CR12] Constantinescu T, Lungu CN (2021) Anticancer activity of natural and synthetic chalcones. Int J Mol Sci 22(21):11306. 10.3390/ijms222111306 34768736 10.3390/ijms222111306 PMC8582663

[CR13] Crossley RM, Johnson S, Tsingos E, Bell Z, Berardi M, Botticelli M, Braat QJS, Metzcar J, Ruscone M, Yin Y, Shuttleworth R (2024) Modeling the extracellular matrix in cell migration and morphogenesis: a guide for the curious biologist. Front Cell Dev Biol 1:1354132. 10.3389/fcell.20241354132 10.3389/fcell.2024.1354132 PMC10940354 38495620

[CR14] De Sá RE, Araújo GS, Silva JM, Aguiar FP, Silva VL, Silva DAD (2023) Chemical composition, antibacterial and cytotoxic activities of essential oil obtained from aerial parts of *Aeschynomene denticulata* rudd. Biocatal Agric Biotechnol 54:102908

[CR15] De Sá RE, de Araújo GS, Machado FDS, Souza JMT, Barros AB, Pinto FDCL, Agostinho JDL, Ayala AP, MarinhoFilho JDB, Pessoa ODL, Araújo AJ (2024) Withaphysalin derivatives from *Iochroma arborescens* induce antiproliferative and antimigratory activities *in vitro*. Planta Med 90:938–948. 10.1055/a-2381-5060 39159664 10.1055/a-2381-5060

[CR16] De Souza PS, Bibá GCC, Melo EDDN, Muzitano MF (2022) Chalcones against the hallmarks of cancer: a mini-review. Nat Prod Res 36:4809–4826. 10.1080/14786419.20212000980 34865580 10.1080/14786419.2021.2000980

[CR17] Eloraby DAI, El-Gayar SF, El-Bolok AH, Ammar SG, ElShafei MM (2024) In vitro assessment of the cytotoxic effect of 5-fluorouracil, thymoquinone and their combination on tongue squamous cell carcinoma cell line. Asian Pac J Cancer Prev 25(6):2169. 10.31557/APJCP.2024.25.6.2169 38918680 10.31557/APJCP.2024.25.6.2169 PMC11382834

[CR18] Eren AD, Lucassen AWA, Tuvshindorj U, Truckenmüller R, Giselbrecht S, Eren ED, Tas MO, Sudarsanam P, de Boer J (2022) Cells dynamically adapt to surface geometry by remodeling their focal adhesions and actin cytoskeleton. Front Cell Dev Biol 10:863721. 10.3389/fcell.2022.863721 35721512 10.3389/fcell.2022.863721 PMC9203963

[CR19] Fathi EM, Sroor FM, Mahrous KF, Mohamed MF, Mahmoud K, Emara M, Elwahy AHM, Abdelhamid IA (2021) Design, synthesis, *in silico* and *in vitro* anticancer activity of novel bis-furanyl-chalcone derivatives linked through alkyl spacers. ChemistrySelect 6(24):6202–6211. 10.1002/slct.202100884

[CR20] Fatima J, Fatima E, Mehmood F, Ishtiaq I, Khan MA, Khurshid HMS, Kashif M (2024) Comprehensive analysis of oral squamous cell carcinomas: clinical, epidemiological, and histopathological insights with a focus on prognostic factors and survival time. Cureus 16:e54394. 10.7759/cureus.54394 38505442 10.7759/cureus.54394 PMC10949903

[CR21] Ferrari IV, Patrizio P (2021) Development and validation molecular docking analysis of human serum albumin (HSA). bioRxiv. 10.1101/2021.07.09.451789

[CR22] Gasteiger J, Marsili M (1980) Iterative partial equalization of orbital electronegativity—a rapid access to atomic charges. Tetrahedron 36:3219–3228. 10.1016/0040-4020(80)80168-2

[CR23] Gerstberger S, Jiang Q, Ganesh K (2023) Metastasis Cell 186:1564–1579. 10.1016/j.cell.2023.03003 37059065 10.1016/j.cell.2023.03.003 PMC10511214

[CR24] Hamidi H, Ivaska J (2019) Every step of the way: integrins in cancer progression and metastasis. Nat Rev Cancer 19:179. 10.1038/s41568-019-0112-1 30705430 10.1038/s41568-019-0112-1

[CR25] Hanahan D (2022) Hallmarks of cancer: new dimensions. Cancer Discov 12:31–46. 10.1158/2159-8290CD-21-1059 35022204 10.1158/2159-8290.CD-21-1059

[CR26] Haque S, Karivedu V, Riaz MK, Choi D, Roof L, Hassan SZ, Zhu Z, Jandarov R, Takiar V, Tang A, Wise-Draper T (2019) High-risk pathological features at the time of salvage surgery predict poor survival after definitive therapy in patients with head and neck squamous cell carcinoma. Oral Oncol 88:9–15. 10.1016/j.oraloncology.2018.11010 30616803 10.1016/j.oraloncology.2018.11.010 PMC6327963

[CR27] He L, Wan M, Yang X, Meng H (2025) Distant metastasis of oral squamous cell carcinoma: immune escape mechanism and new perspectives on treatment. Discov Oncol 16:257. 10.1007/s12672-025-01997-3 40024975 10.1007/s12672-025-01997-3 PMC11872995

[CR28] Janiszewska M, Primi MC, Izard T (2020) Cell adhesion in cancer: beyond the migration of single cells. J Biol Chem 295:2495–2505. 10.1074/jbc.REV119.007759 31937589 10.1074/jbc.REV119.007759 PMC7039572

[CR29] Jansson-Löfmark R, Hjorth S, Gabrielsson J (2020) Does *in vitro* potency predict clinically efficacious concentrations? Clin Pharmacol Ther 108:298–305. 10.1002/cpt.1836 32275768 10.1002/cpt.1846 PMC7484912

[CR30] Kim IS, Yang WS, Kim CH (2023) Physiological properties, functions, and trends in the matrix metalloproteinase inhibitors in inflammation-mediated human diseases. Curr Med Chem 30:2075–2112. 10.2174/0929867329666220823112731 36017851 10.2174/0929867329666220823112731

[CR31] Lambert AW, Zhang Y, Weinberg RA (2024) Cell-intrinsic and microenvironmental determinants of metastatic colonization. Nat Cell Biol 1:1–11. 10.1038/s41556-024-01409-8 10.1038/s41556-024-01409-8 38714854

[CR32] Lenci E, Cosottini L, Trabocchi A (2021) Novel matrix metalloproteinase inhibitors: an updated patent review (2014–2020). Expert Opin Ther Pat 31:509–523. 10.1080/135437762021 33487088 10.1080/13543776.2021.1881481

[CR33] Li M, Xi N, Wang Y, Liu L (2021) Atomic force microscopy for revealing micro/nanoscale mechanics in tumor metastasis: from single cells to microenvironmental cues. Acta Pharmacol Sin 42:323–339. 10.1038/s41401-020-00525-z 32807839 10.1038/s41401-020-0494-3 PMC8027022

[CR34] Liang G, Shao L, Wang Y, Zhao C, Chu Y, Xiao J, Zhao YX, Yang S (2009) Exploration and synthesis of curcumin analogues with improved structural stability both in vitro and in vivo as cytotoxic agents. Bioorg Med Chem 17:2623–2631. 10.1016/j.bmc.2008.10044 19243951 10.1016/j.bmc.2008.10.044

[CR35] Lima LTF, Ganzella FAO, Cardoso GC, Pires VDS, Chequin A, Santos GL, Braun-Prado K, Galindo CM, Braz Junior O, Molento MB, Acco A, Adami ER, Costa ET, Franco CRC, Klassen G, Ramos EAS (2023) L-carvone decreases breast cancer cells adhesion, migration, and invasion by suppressing FAK activation. Chem Biol Interact 378:110480. 10.1016/j.cbi.2023.110480 37059214 10.1016/j.cbi.2023.110480

[CR36] Livak KJ, Schmittgen TD (2001) Analysis of relative gene expression data using real-time quantitative pcr and the 2(-delta delta c(t)) method. Methods 25:402–408. 10.1006/meth.2001.1262 11846609 10.1006/meth.2001.1262

[CR37] Mancini A, Gentile MT, Pentimalli F, Cortellino S, Grieco M, Giordano A (2024) Multiple aspects of matrix stiffness in cancer progression. Front Oncol 14:1406644. 10.3389/fonc.20241406644 39015505 10.3389/fonc.2024.1406644 PMC11249764

[CR38] Modzelewska A, Pettit C, Achanta G, Davidson NE, Huang P, Khan SR (2006) Anticancer activities of novel chalcone and bis-chalcone derivatives. Bioorg Med Chem 15:3491–3495. 10.1016/j.bmc.2006.01.003 10.1016/j.bmc.2006.01.003 16434201

[CR39] Morris GM, Huey R, Olson AJ (2008) Using autodock for ligand-receptor docking. Curr Protoc Bioinform 24:8–14. 10.1002/0471250953 10.1002/0471250953.bi0814s24 19085980

[CR40] Mosmann T (1983) Rapid colorimetric assay for cellular growth and survival: application to proliferation and cytotoxicity assays. J Immunol Methods 65:55–63. 10.1016/0022-1759(83)90303-4 6606682 10.1016/0022-1759(83)90303-4

[CR41] Nian C, Gan X, Liu Q, Wu Y, Kong M, Zhang P, Jin M, Dong Z, Li W, Wang L, He W (2024) Synthesis and anti-gastric cancer activity by targeting FGFR1 pathway of novel asymmetric bis-chalcone compounds. Curr Med Chem 31(39):6521–6541. 10.2174/0109298673298420240530093525 10.2174/0109298673298420240530093525 38847254

[CR42] Olender D, Kujawski J, Skóra B, Baranowska-Wójcik E, Sowa-Kasprzak K, Pawełczyk A, Zaprutko L, Szwajgier KA (2024) Bis-chalcones obtained via one-pot synthesis as anti-neurodegenerative agents and their effect on the HT-22 cell line. Heliyon 10:e37147. 10.1016/j.heliyon.2024.e31747 39286165 10.1016/j.heliyon.2024.e37147 PMC11403034

[CR43] Pang X, He X, Qiu Z, Zhang H, Xie R, Liu Z, Gu Y, Zhao N, Xiang Q, Cui Y (2023) Targeting integrin pathways: mechanisms and advances in therapy. Signal Transduct Target Ther 8:1. 10.1038/s41392-022-01250-x 36588107 10.1038/s41392-022-01259-6 PMC9805914

[CR44] Pearson GW (2019) Control of invasion by epithelial-to-mesenchymal transition programs during metastasis. J Clin Med 8:646. 10.3390/jcm8050646 31083398 10.3390/jcm8050646 PMC6572027

[CR45] Pereira R, Silva AMS, Ribeiro D, Silva VLM, Fernandes E (2023) Bis-chalcones: a review of synthetic methodologies and anti-inflammatory effects. Eur J Med Chem 5:115280. 10.1016/j.ejmech.2023115280 10.1016/j.ejmech.2023.115280 36966653

[CR46] Pettersen EF, Goddard TD, Huang CC, Couch GS, Greenblatt DM, Meng EC, Ferrin TE (2004) UCSF Chimera – a visualization system for exploratory research and analysis. J Comput Chem 25:1605–1612. 10.1002/jcc.20084 10.1002/jcc.20084 15264254

[CR47] Rajendran P (2024) Unveiling the power of flavonoids: a dynamic exploration of their impact on cancer through matrix metalloproteinases regulation. Biomedicine (Taipei) 14:12–28. 10.37796/2211-80391447 38939095 10.37796/2211-8039.1447 PMC11204124

[CR48] Repasky MP, Shelton HA, Ebe K, Sastry GM, Ebe J, Abel R, Friesner RA (2012) Docking performance of the glide program as evaluated on the Astex and DUD datasets: a complete set of glide SP results and selected results for a new scoring function integrating WaterMap and glide. J Comput Aided Mol Des 26:787–799. 10.1007/s10822-012-9599-4 22576241 10.1007/s10822-012-9575-9

[CR49] Rocha JA, Rego NC, Carvalho BT, Silva FI, Sousa JA, Ramos RM, Passos ING, Moraes J, Leite JRSA, Lima FC (2018) Computational quantum chemistry, molecular docking, and ADMET predictions of imidazole alkaloids of *Pilocarpus microphyllus* with schistosomicidal properties. PLoS One 13:e0198476. 10.1371/journal.pone.0198476 29944674 10.1371/journal.pone.0198476 PMC6019389

[CR50] Sati P, Sharma E, Dhyani P, Attri DC, Rana R, Kiyekbayeva L, Büsselberg D, Samuel SM, Sharifi-Rad J (2024) Paclitaxel and its semi-synthetic derivatives: comprehensive insights into chemical structure, mechanisms of action, and anticancer properties. Eur J Med Res 29(1):90. 10.1186/s40001-024-01657-2 38291541 10.1186/s40001-024-01657-2 PMC10826257

[CR51] Sharma A, Chakravarti B, Gupt MP, Siddiqui JA, Konwar R, Tripathi RP (2010) Synthesis and anti breast cancer activity of biphenyl based chalcones. Bioorg Med Chem 18:4711–4720. 10.1016/j.bmc.2010.05.015 20605470 10.1016/j.bmc.2010.05.015

[CR52] Shi X, Wang X, Yao W, Shi D, Shao X, Lu Z, Chai Y, Song J, Tang W, Wang X (2024) Mechanism insights and therapeutic intervention of tumor metastasis: latest developments and perspectives. Signal Transduct Target Ther 9:192. 10.1038/s41392-024-01885-2 39090094 10.1038/s41392-024-01885-2 PMC11294630

[CR53] Sung H, Ferlay J, Siegel R, Laversanne J, Soerjomataram I, Jemal A, Bray F (2021) Global cancer statistics 2020: GLOBOCAN estimates of incidence and mortality worldwide for 36 cancers in 185 countries. CA Cancer J Clin 71:209–249. 10.3322/caac.21660 33538338 10.3322/caac.21660

[CR54] Trott O, Olson AJ (2010) Autodock vina: improving the speed and accuracy of docking with a new scoring function, efficient optimization, and multithreading. J Comput Chem 31:455–461. 10.1002/jcc.21334 19499576 10.1002/jcc.21334 PMC3041641

[CR55] Welch DR, Hurst DR (2019) Defining the hallmarks of metastasis. Can Res 79:3011–3027. 10.1158/0008-5472CAN-19-0458 10.1158/0008-5472.CAN-19-0458 PMC6571042 31053634

[CR56] Xiao H, Chen Y, Alnaggar M (2019) Silver nanoparticles induce cell death of colon cancer cells through impairing cytoskeleton and membrane nanostructure. Micron 126:102750. 10.1016/j.micron.2019102750 31522088 10.1016/j.micron.2019.102750

[CR57] Yang J, Mu WW, Liu GY (2020) Synthesis and evaluation of the anticancer activity of bis-chalcone analogs in human lung carcinoma (A549) cell line. Eur J Pharmacol 5:173396. 10.1016/j.ejphar.2020173396 10.1016/j.ejphar.2020.173396 32798508

[CR58] Zhang S, Li T, Zhang L, Wang X, Dong H, Li L, Fu D, Li Y, Zi X, Liu H-M, Zhang Y, Xu H, Jin C-Y (2017) A novel chalcone derivative S17 induces apoptosis through ROS-dependent DR5 up-regulation in gastric cancer cells. Sci Rep 7:10400. 10.1038/s41598-017-10400-3 28852176 10.1038/s41598-017-10400-3 PMC5575266

[CR59] Zhao C, Hou X, Peng Z, Sun X, Li E, Yang H, Lu Y, Zhu L (2020) Estrogen receptor alpha depletion affects the biomechanical properties and cytoskeleton rearrangements in breast cancer cells. Biochem Biophys Res Commun 291:30296–30305. 10.1016/j.bbrc.2020.02030 10.1016/j.bbrc.2020.02.030 32081423

[CR60] Zhuang C, Zhang W, Sheng C, Zhang W, Xing C, Miao Z (2017) Chalcone: a privileged structure in medicinal chemistry. Chem Rev 117:7762–7810. 10.1021/acs.chemrev.7b00020 28488435 10.1021/acs.chemrev.7b00020 PMC6131713

