# Supplementary material for: Dual inhibition of MMP-2 and actin dynamics by a novel bis-chalcone: an anticancer strategy for oral squamous cell carcinoma
Source: Naunyn Schmiedebergs Arch Pharmacol. 2026 Feb 21;399(8):11643–58. doi: 10.1007/s00210-026-05050-0 (PMC13269452; doi:10.1007/s00210-026-05050-0)
Supplement: Supplementary file 1 — Supplementary file1 (DOCX 6464 KB) [file 210_2026_5050_MOESM1_ESM.docx]

**Supplementary Material**

**Dual inhibition of MMP-2 and actin dynamics by a novel bis-chalcone: an anticancer strategy for oral squamous cell carcinoma**

*Rodrigo Elísio de Sá^a^, Bruna Oliveira de Almeida^b^, Marcelo da Costa Mota^e^, Matheus Pedrosa de Oliveira^a^, Anali Del Milagro Bernabe Garnique^b^, Keli Lima^b^,* *Aline Bernardes Valeze^c^, Jefferson Almeida Rocha^e^, Caridad Noda Pérez^d^, João Agostinho Machado Neto^b^, Letícia Veras Costa Lotufo^b^, José Delano Barreto Marinho Filho^a^, Ana Jérsia Araújo^a*^*

*^a^Laboratório de Cultura de Células do Delta (LCCDelta), Universidade Federal Delta do Parnaíba, UFDPar, Parnaíba, PI, Brazil*

*^b^Departamento de Farmacologia, Instituto de Ciências Biomédicas, Universidade de São Paulo, USP, São Paulo, SP, Brazil*

*^c^Departamento de Ensino, Instituto Federal de Educação, Ciência e Tecnologia de Mato Grosso - IFMT, Cuiabá, MT, Brazil*

***^d^*** *Laboratório de Sínteses Orgânica e Catálise, Instituto de Química, Universidade Federal do Goiás, UFG, Goiânia, GO, Brazil*

*^e^Grupo de Pesquisa em Química Medicinal e Biotecnologia, QUIMEBIO, Universidade Federal do Maranhão, UFMA, São Bernardo, MA, Brazil*

** Corresponding author*

Ana Jérsia Araújo, PhD. Laboratório de Cultura de Células do Delta (LCCDelta), Universidade Federal do Delta de Parnaíba, UFDPar, 64202-020, Parnaíba, Brazil. Tel: +55 86 99439 9223. E-mail address: [anajersia@ufdpar.edu.br](mailto:anajersia@ufdpar.edu.br). ORCID: 0000-0001-7182-7097

**General experimental procedures and cell lines used**

Infrared (IR) spectra were recorded on a Bomem M102 spectrometer (Bomem Inc., Vanier, Quebec, Canada). ¹H NMR spectra were acquired using a Bruker Avance III 500 MHz spectrometer (Bruker Optik GmbH, Ettlingen, Germany). Melting points were determined with a melting point apparatus (Karl Kolb GmbH & Co. Dreieich, Germany). Elemental analyses (C, H) were performed using a Thermo Scientific Flash 2000 CHNS-O analyzer equipped with a thermal conductivity detector (TCD). Cell cultures were maintained in Dulbecco’s Modified Eagle Medium (DMEM; Sigma®) supplemented with 10% fetal bovine serum (FBS; GIBCO®) and 1% penicillin–streptomycin (Sigma®). Dimethyl sulfoxide (DMSO; Exodocientífica®) was used as the vehicle for compound dilution. Doxorubicin, type I collagen, crystal violet, and other salts and reagents were obtained from Sigma-Aldrich (St. Louis, MO, USA). Trypsin–EDTA 0.5% (Cultilab®), May–Grünwald (RenyLab), Giemsa dye (Chromoline), MTT (Invitrogen®), trypan blue (Dinâmica®), and Matrigel (Collaborative Biomedical Products) were also commercially purchased. Light microscopy and staining were performed using a Rapid Panoptic Staining Kit (Laborclin®) and a Primo Star light microscope (ZEISS), respectively. Colony images were captured using a G:BOX Chemi XRQ imaging system (Syngene) and analyzed with ImageJ software (v1.45s). Spheroid images were acquired using a digital inverted light microscope (EVOS AME-3302). Total RNA was extracted with TRIzol reagent (Thermo Fisher Scientific, San Jose, CA, USA). Complementary DNA (cDNA) was synthesized using the High-Capacity cDNA Reverse Transcription Kit (Thermo Fisher Scientific). Quantitative PCR (qPCR) was performed on an ABI 7500 Sequence Detection System (Thermo Fisher Scientific) using primers obtained from Integrated DNA Technologies (IDT, Coralville, IA, USA). For Western blotting, nitrocellulose membranes were obtained from GE Healthcare (Milwaukee, WI, USA). Primary antibodies against GAPDH (#5174), pan-actin (#4968), and MMP-2 (#40994), as well as HRP-conjugated secondary anti-rabbit (#7074) and anti-mouse (#7076), were purchased from Cell Signaling Technology (Beverly, MA, USA). Chemiluminescent detection was performed using the SuperSignal™ West Dura substrate (Thermo Fisher Scientific), and images were acquired with a G:BOX Chemi XX6 imaging system (Syngene). Immunofluorescence staining was conducted using anti-α-tubulin Alexa Fluor™ 488 conjugate (Thermo Fisher Scientific, cat. no. 53-4502-82) and Alexa Fluor™ 633–phalloidin. Slides were mounted with ProLong™ Gold Antifade Mountant with DAPI (Thermo Fisher Scientific). Images were captured with a Lionheart FX Automated Fluorescence Microscope (BioTek Instruments Inc., Winooski, VT, USA). The HSC-3, SCC-4, and CAL-27 human tongue squamous cell carcinoma cell lines, as well as HS-5 stromal and HaCaT keratinocyte cell lines, were kindly provided by the University of São Paulo (USP) and the Oswaldo Cruz Foundation (Fiocruz), respectively. Statistical analyses were performed using GraphPad Prism software (v.9.0; GraphPad Software Inc., San Diego, CA, USA).

**Supplemental Table 1** Active site coordinates and RMSD values obtained from the re-docking

| Complex: Co-crystallized Ligand/Protein | RMSD (Å) | Grid center coordinates | Grid box size |
| --- | --- | --- | --- |
| ADP/2ZWH | 0.85 | X = 17.35  Y = -4.624  Z = 3.923 | 20 x  36 y  22 z |
| L2U/8H78 | 1.83 | X = 28.590  Y = 24.391  Z = -8.297 | 52 x  70 y  42 z |
| B9Z/6ESM | 0.84 | X = 0.826  Y = 50.366  Z = 19.911 | 18 x  34 y  36 z |

ADP: Adenosine-5'-diphosphate (CID: PubChem 6022); 2ZWH: F-actin; L2U: CHEMBL5412341 (CID: PubChem 166451148); 8H78: Matrix metalloproteinase-2 (MMP-2); B9Z: (2~{S})-2-[2-[4-(4-methoxyphenyl) phenyl] sulfanylphenyl] pentanedioic acid (CID: PubChem 133084111); 6ESM: Matrix metalloproteinase-9 (MMP-9).

| Gene^1^ | Sequence |
| --- | --- |
| *MMP-2* | FW: TAT GGC TTC TGC CCT GAG AC  RV: CAC ACC ACA TCT TTC CGT CA |
| *MMP-9* | FW: CGA ACT TTG ACA GCG ACA AG  RV: CGG CAC TGA GGA ATG ATC TA |
| *GAPDH* | FW: CCA CCC ATG GCA AAT TCC  RV: GAT GGG ATT TCC ATT GAT GAC A |

**Supplemental Table 2** PCR results for genes related to endopeptidases after exposure to B2OCH_3_ in HSC-3 cells

^1^Genes are reported according to Human Genome Organization (HUGO) Gene Nomenclature Committee (HGNC).

Abbreviations: FW, forward; RV, reverse.

**Supplemental Figure 1** Infrared (IR) spectrum of compound B2OCH₃.

The infrared (IR) spectrum of B2OCH3 shows characteristic absorption bands at 3413 cm⁻¹ (O–H stretching vibration), 2940–2840 cm⁻¹ (C–H stretching of methyl and methoxy groups), and 1710 cm⁻¹ (C=O stretching vibration). The bands at 1607, 1515, and 1465 cm⁻¹ correspond to aromatic C=C stretching, while those at 1354 and 1245 cm⁻¹ are attributed to C–O stretching vibrations. Additional signals at 1170, 1102, and 1020 cm⁻¹ are consistent with ether (C–O–C) and methoxy functionalities. The region below 900 cm⁻¹ (notably at 875, 820, and 750 cm⁻¹) exhibits aromatic out-of-plane C–H bending modes, confirming the presence of a substituted aromatic ring. The overall spectral profile supports the presence of hydroxyl, carbonyl, and methoxy groups in the structure of B2OCH_3_.

**Supplemental Figure 2.** Infrared (IR) spectrum of compound B2OCH₃.


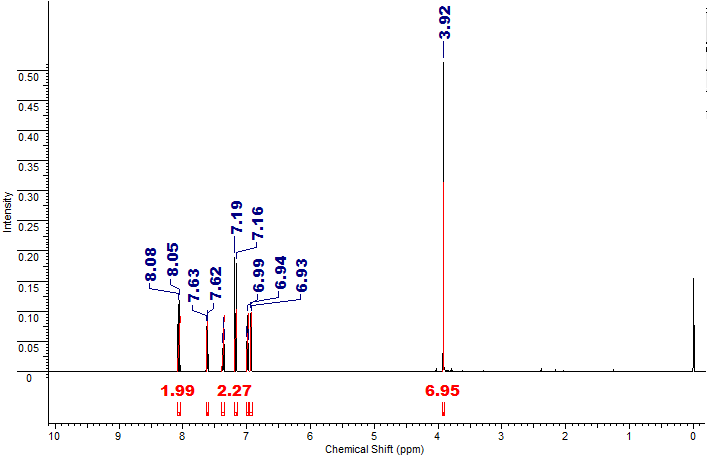


The ¹H NMR spectrum of B2OCH₃ exhibits characteristic aromatic proton signals between δ 6.93–8.08 ppm, consistent with a substituted aromatic system. Multiplets at δ 7.16-7.62 ppm correspond to protons of the aromatic rings, while singlets at δ 1.99 and 2.27 ppm are assigned to methyl groups attached to the aromatic moiety. A singlet at δ 3.92 ppm is attributed to methoxy (–OCH₃) protons. The signal observed at δ 6.95 ppm corresponds to an olefinic proton, typical of conjugated systems such as α, β-unsaturated carbonyl compounds. The overall spectral pattern supports the proposed structure of B2OCH₃, confirming the presence of aromatic, methoxy, methyl, and olefinic protons.

**Supplemental Figure 3** Dose-response curves of B2OCH₃ cytotoxicity kinetics after 24, 48, and 72 h of treatment in HS-5 cells (n = 3) (mean ± SEM).

Results are expressed as a percentage of the mean ± standard error of the mean (SEM). Data represent the average of three independent experiments. The selectivity index (SI) was calculated as the ratio of the IC₅₀ value of the non-tumor HS-5 cell line to that of the tumor HSC-3 cell line, as described in the main manuscript.

**Supplemental Table 3** IC_50_ values of the positive control (Doxorubicin) in HSC-3 and HS5 cells after 24, 48, and 72 h of incubation

|  | **IC_50_ (µM)**  **95% Confidence interval** | |
| --- | --- | --- |
| **Time** | **HSC-3** | **HS-5** |
| **24h** | 0.29  (0.21 – 0.38) | 0.62  (0.5 – 0.9)  0.53  (0.45 – 0.67) |
| **48h** | 0.25  (0.21 – 0.30) |  |
| **72h** | 0.21  (0.20 – 0.29) | 0.33  (0.25 – 0.47) |

The half-maximal inhibitory concentration (IC_50_) values were calculated using nonlinear regression with GraphPad Prism Software version 8.0. The 95% confidence intervals were obtained from at least three independent experiments performed in triplicate. Doxorubicin was used as a positive control.

**Supplemental Figure 4.** High-resolution immunofluorescence images showing cytoskeletal alterations in HSC-3 cells treated with B2OCH₃

Representative immunofluorescence images showing nuclei (DAPI, blue), microtubules (α-tubulin, green), and actin filaments (F-actin, phalloidin, red) in HSC-3 cells after exposure to B2OCH₃ (0.5 µM) for 3 and 6 h, compared with untreated control cells. Arrows indicate cytoskeletal alterations. A marked reduction in phalloidin signal intensity and disorganization of actin filaments and filopodia were observed in treated cells, indicating cytoskeletal disruption. Images were acquired using a Lionheart FX Automated Fluorescence Microscope (BioTek Instruments, USA). Scale bar = 30 µm.

**Supplemental Figure 5.** Two-dimensional molecular interaction maps of B2OCH₃


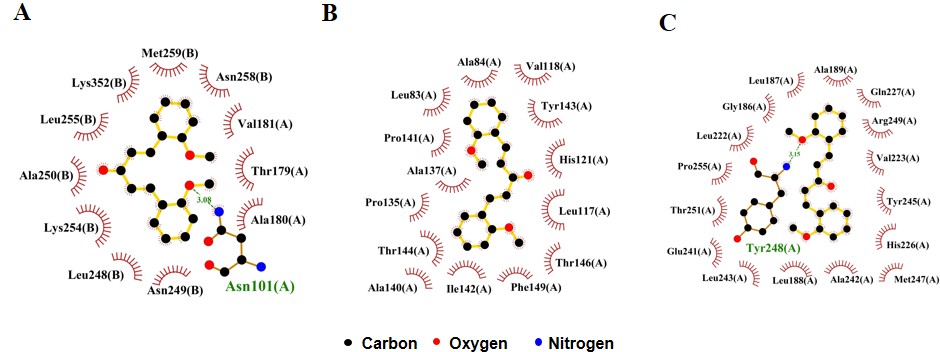


Two-dimensional interaction diagrams illustrating the predicted binding of B2OCH₃ to actin (A), MMP-2 (B), and MMP-9 (C), as obtained from molecular docking analyses. The diagrams depict the amino acid residues surrounding the ligand within the binding pocket and the main non-covalent interactions stabilizing each complex. Hydrogen bond interactions are indicated by green dashed lines with corresponding distances (Å), while red arcs represent hydrophobic contacts. Carbon, oxygen, and nitrogen atoms are shown in black, red, and blue, respectively.

**Supplemental Figure 6.** Representative full-length Western blot images of GAPDH, MMP-2, and pan-actin proteins


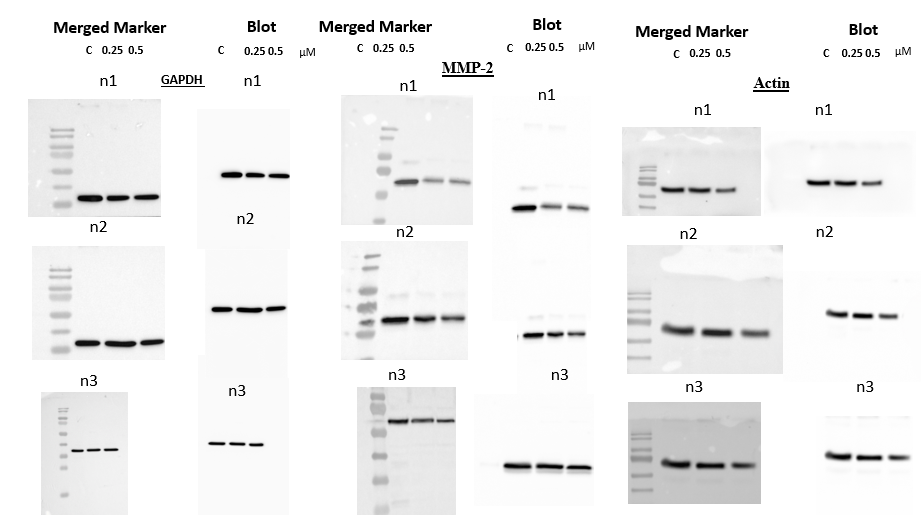


Protein expression levels of GAPDH (37 kDa), MMP-2 (64/72 kDa), and pan-actin (42 kDa) were evaluated by Western blot after 24 h of treatment with B2OCH3 at concentrations of 0.25 and 0.5 µM. Each lane corresponds to independent biological replicates (n1, n2, n3). The control group (C) represents untreated cells. Equal protein loading was confirmed by probing the membranes with anti-GAPDH antibodies. A concentration-dependent decrease in MMP-2 and pan-actin expression was observed compared with the control group, while GAPDH levels remained constant, confirming sample integrity and normalization. *Note:* Representative blots are shown from three independent experiments. Quantitative densitometric analyses are presented in the main figures (see Fig. 6E, F). High-resolution, uncropped blots are provided here for transparency.
